# Winged Threat on the Offensive: A Literature Review Due to the First Identification of *Aedes japonicus* in Poland

**DOI:** 10.3390/v16050703

**Published:** 2024-04-29

**Authors:** Marcin Gierek, Gabriela Ochała-Gierek, Andrzej Józef Woźnica, Grzegorz Zaleśny, Alicja Jarosz, Paweł Niemiec

**Affiliations:** 1 Center for Burns Treatment, 41-100 Siemianowice Śląskie, Poland; marcin.gierek@clo.com.pl; 2 Gierek Clinic, ul. Zielona 2, 43-450 Ustroń, Poland; g.ochala@wp.pl; 3 Institute of Environmental Biology, Wrocław University of Environmental and Life Sciences, Kożuchowska St. 5B i 7A, 51-631 Wrocław, Poland; grzegorz.zalesny@upwr.edu.pl; 4 Department of Biochemistry and Medical Genetics, School of Health Sciences, Medical University of Silesia in Katowice, ul. Medykow 18, 40-752 Katowice, Poland; pniemiec@sum.edu.pl

**Keywords:** *Aedes japonicus*, invasive mosquito, vector-borne diseases, disease transmission, chikungunya, dengue, West Nile virus, Zika virus, vaccine, public health

## Abstract

Genetic studies preceded by the observation of an unknown mosquito species in Mikołów (Poland) confirmed that it belongs to a new invasive species in Polish fauna, *Aedes japonicus* (Theobald, 1901), a known vector for numerous infectious diseases. *Ae. japonicus* is expanding its geographical presence, raising concerns about potential disease transmission given its vector competence for chikungunya virus, dengue virus, West Nile virus, and Zika virus. This first genetically confirmed identification of *Ae. japonicus* in Poland initiates a comprehensive review of the literature on *Ae. japonicus*, its biology and ecology, and the viral infections transmitted by this species. This paper also presents the circumstances of the observation of *Ae. japonicus* in Poland and a methodology for identifying this species.

## 1. Introduction

Globalization and climate change contribute to the migration of invasive insect species, which also affects the territory of Poland [1]. Urban areas, with their landscape structure, lack of natural regulatory mechanisms, and abundance of potential substitute microhabitats, are particularly vulnerable to biodiversity loss and susceptible to colonization [2]. The intensification of colonization in Europe by species potentially dangerous to human health and life, such as mosquitoes (family: Culicidae; order: Diptera), which are vectors of many infectious diseases, raises the greatest concerns. The observation of *Aedes japonicus* (Theobald, 1901), one of several invasive mosquito species in Europe, occurred in Mikołów (Upper Silesia) in October 2023. Species identification was confirmed by genetic studies, prompting the authors to conduct the current literature review.

The main aim of the current work is to present current data on viral diseases transmitted by *Aedes japonicus*, as well as contemporary therapeutic and preventive strategies. The Medline (PubMed) and Scopus databases were searched by two independent researchers (M.G.; P.N.) using the following terms: “*Aedes japonicus*” or “Asian bush mosquito” and “virus”, “vector”, “host”, “dengue”, “Zika”, “chikungunya”, and “West Nile fever”. A bibliography of the current status of vaccinations for specific diseases was also obtained from the same sources. Relevant original and review studies published in English up to December 2023 were screened and selected. The actual literature review is preceded by a section in which we present the circumstances of observing *Ae. japonicus* in Poland, along with the adopted species identification methodology.

## 2. Observation of *Ae. japonicus* in Poland and Its Identification

**Figure 1:**
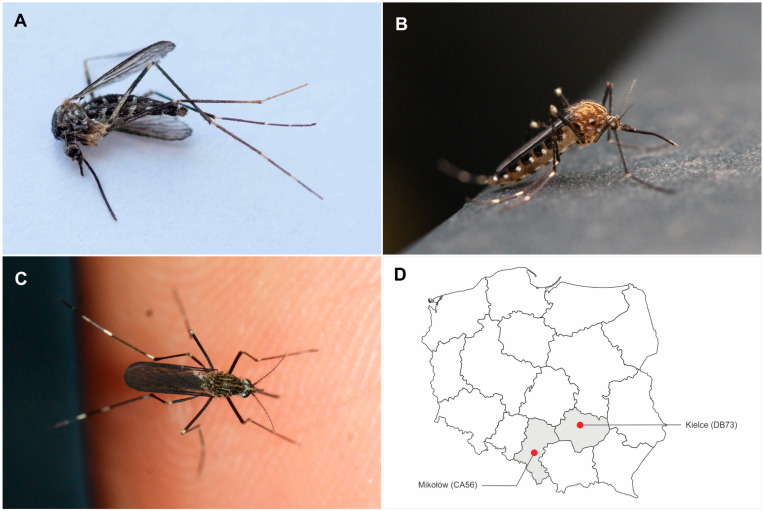
Observed individuals of *Ae. japonicus* in Poland. (**A**) CA56 Mikołów, 4.X.2023; (**B**) DB73 Kielce, 30.X.2022; (**C**) DB73 Kielce, 21.X.2023; (**D**) locations of observations on a map of Poland.

The first genetically confirmed observation of *Ae. japonicus* in Poland concerns an individual found in a house located in Mikołów, Silesian Voivodeship (CA56, 4.X.2023, leg. G. Ochała-Gierek and M. Gierek) (Figure 1A). The hand-picking method, sometimes known as the “One-Hand Snatch” technique, was used to collect the mosquito. After being preserved in 95% ethanol, the specimen was subjected to genetic analysis, confirming an earlier determination based on morphological characteristics [3,4,5,6,7,8]. On 14 October 2023, in a nearby forest, numerous mosquito individuals resembling the initial specimen were observed (CA56 Mikołów, leg. M. Gierek). Six of them were manually captured (hand-picking method), preserved, and subjected to further identification. All were morphologically identified as *Ae. japonicus* individuals, with one selected for genetic analysis, confirming its membership in this species. Additional information regarding the occurrence of *Ae. japonicus* in Poland, not confirmed by genetic studies, comes from the online citizen science project iNaturalist (as part of the Mosquito Vectors Project). Observations involved two females photographed in a private garden in Kielce, Świętokrzyskie Voivodeship (DB73, 30.X.2022, 1 ex., 21.X.2023, 1 ex., leg. K. Barszcz) (Figure 1B,C) [9]. Figure 1D shows the location of both observation sites on a map of Poland.

The identification of the analyzed material (collected samples and pictures) was performed using Moskeytool version 2.2 [3] and based on the literature [4,5,6,7,8]. The characteristic features of *Ae*. *japonicus* are pleural and mesonotal setulae and body coloration (groups of black and white pleural setulae, especially obvious in fresh specimens). The scutum of the thorax is covered with golden-yellow lyre stripes; the anterior dorsocentral ones are longer than those in a related species, *Aedes koreicus* and extend visibly beyond half of the scutum (Figure 1C). The hind tarsomeres are provided by three whitish cross-bands on the hind legs. In *Ae*. *koreicus*, the anterior dorsocentral stripe is distinctly shorter, ending before the lateral line bend, and the hind legs are characterized by the presence of cross-bands on the hind tarsomere (sometimes with five additional incomplete white ones) [10].

**Figure 2:**
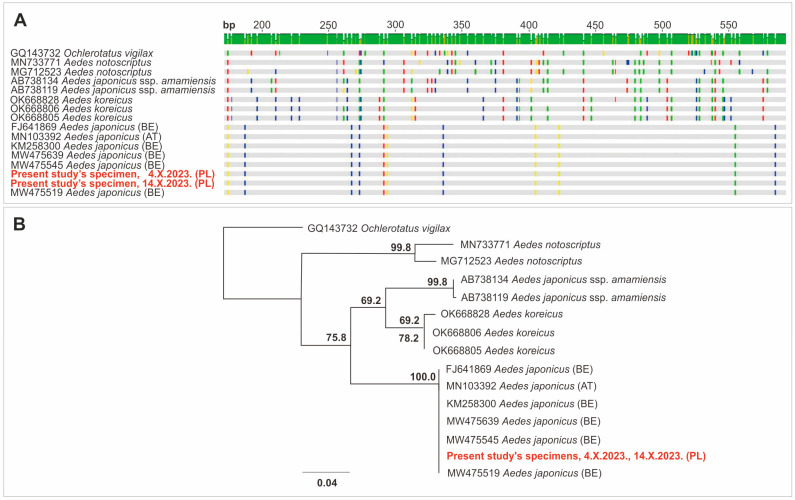
Identification of *Aedes japonicus* (Theobald, 1901) based on sequencing: (**A**) similarity of *COI* mtDNA sequence of two Polish specimens (CA56 Mikołów) to selected sequences of potentially related mosquito species (BLAST, NCBI); (**B**) phylogenetic tree illustrating the taxonomic status of specimens from Poland. Legend: bp, base pairs.

Genetic determination of the species, based on Sanger sequencing, was preceded by DNA isolation and Polymerase Chain Reaction (PCR) amplification. DNA was isolated from two specimens collected in Mikołów (4.X.2023, 14.X.2023) by the use of a commercial kit (DNeasy Blood and Tissue Kit, Qiagen, Hilden, Germany). Next, a conventional PCR reaction was performed with the LCO1490 and HCO 2198 primers [11], partially covering the oxidase cytochrome 1 subunit (*COI*) of mt DNA. The PCR product was purified with Exo-BAP (EurX, Gdańsk, Poland) and sequenced with the same set of primers. Contigs were aligned in Geneious Prime 2023 (Biomatters Ltd., Auckland, New Zealand), and the consensus sequence was BLAST-searched on the NCBI database. Next, selected sequences were aligned, and maximum-likelihood-based phylogenetic analysis was performed with the use of the PhyML plugin [12] implemented in Geneious Prime 2023 (Biomatters Ltd.). A 685 bp sequence of *COI* mtDNA (GenBank Acc. No. PP587562) was obtained, which was completely identical (100% similarity) to sequences of *Ae. japonicus* originating from Belgium and Austria (Figure 2A) and very similar (a two-nucleotide difference) to Italian or Slovenian isolates. The phylogenetic tree (Figure 2B) clearly confirmed the taxonomical status of the isolates and placed them among the *Ae. japonicus* clade; thus, this finding is the first molecular confirmation of the presence of this species in the territory of Poland.

## 3. Occurrence, Expansion and Ecology of *Aedes japonicus*

**Figure 3:**
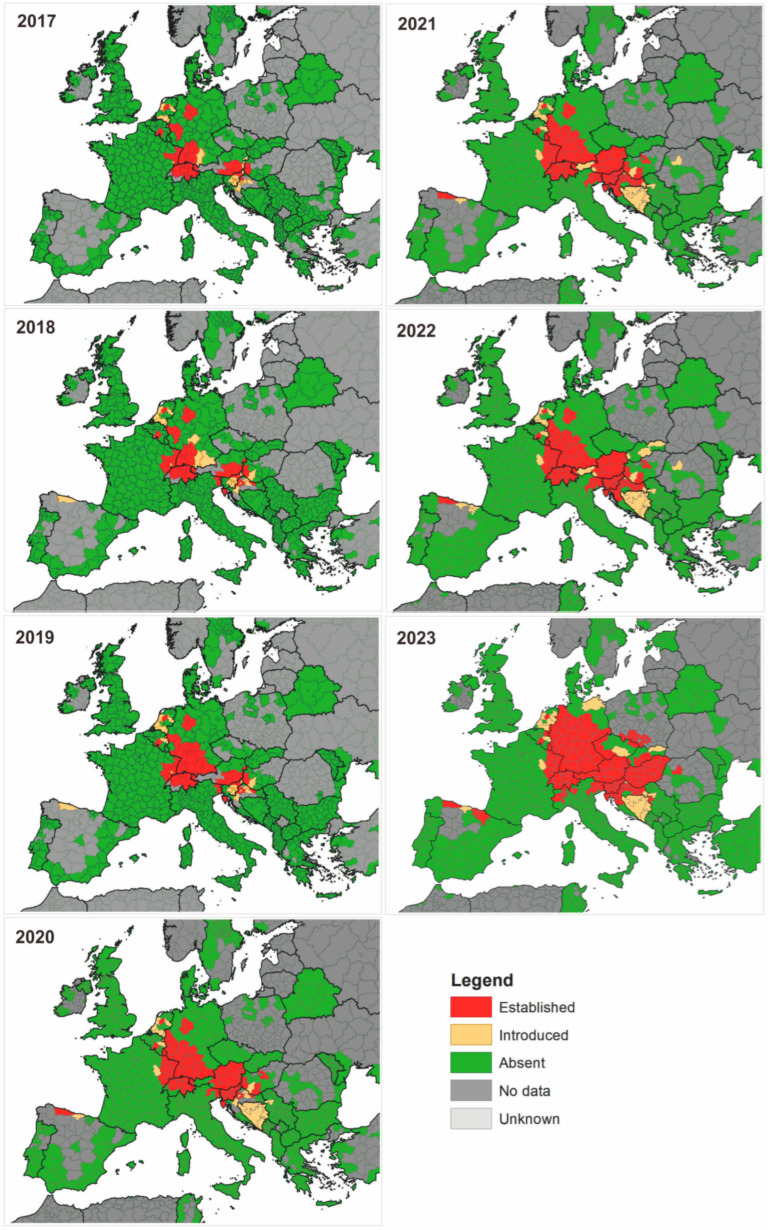
The expansion of *Aedes japonicus* (Theobald, 1901) in Europe over the years 2017–2023. On the basis of [16], accessed online 17 November 2023.

The Asian bush mosquito *Aedes japonicus* (Theobald, 1901), the third invasive mosquito species documented in Europe, has expanded its geographical presence, facilitated by human activities, notably international trade in used tires. *Ae. Japonicus* is natively distributed in Eastern Asia (Taiwan, Hong Kong, and Japan and parts of Russia, China, and the Korean Peninsula) [5]. After its initial detection in France and subsequent reports in Belgium and Switzerland, the species has continued to expand its range in Europe [13,14,15]. According to data for 2023, its occurrence in Europe includes the Netherlands, Germany, the Czech Republic, Slovakia, Hungary, Austria, Switzerland, Liechtenstein, Luxembourg, Italy, Slovenia, Croatia, Spain, and Romania [16]. It has also been introduced to the United States, where it is widely distributed [17]. The presence of *Ae. japonicus* has also been detected in Canada and Hawaii [18,19]. Figure 3 presents the expansion of *Ae. japonicus* in Europe over the years 2017–2023 [16].

The success of its invasion, particularly in the United States, can be attributed to factors such as its adaptability to long-distance dispersal and tolerance to winter temperatures in temperate regions [13,17]. Unlike *Aedes albopictus* (Skuse, 1895), *Ae. japonicus* displays less specialized aquatic habitat requirements, potentially promoting its multiplication and survival: The larvae of *Ae. Japonicus* tolerate a wider range of temperatures and habitats, from natural to small containers. Additionally, *Ae. japonicus* has greater tolerance to lower temperatures, prefers temperate climates, and can survive cold conditions by entering diapause in the egg stage [14]. Concerns have arisen regarding the potential for *Ae. japonicus* to become an invasive species, which may contribute to the transmission of North American arboviruses, as well as the West Nile virus (WNV) [15]. This species has demonstrated a capacity to colonize urban environments, with daytime activity patterns increasing the risk of contact with humans and subsequent disease transmission. Subsequent findings have indicated that *Ae. japonicus* is a vector potential for dengue and chikungunya, both of which have emerged in Europe [20].

Morphologically, adult *Ae. japonicus* exhibits distinct black-and-white patterns, with diagnostic features including yellowish scale lines on the dorsal thorax (scutum) [5]. A DNA-based assay facilitates accurate species differentiation [21]. Freeze and desiccation-resistant eggs enable the species to endure adverse conditions, facilitating transportation in infested containers [22]. *Aedes japonicus* displays adaptability to various aquatic habitats, including rock pools, tires, bird baths, and tree holes [23]. 

The feeding preferences of *Ae. japonicus* lean toward mammalian hosts, with evidence suggesting a preference for humans [24]. Limited information on environmental constraints suggests a potential limitation in habitats with water temperatures exceeding 30 °C [25,26]. This could potentially serve as a limiting factor for its future expansion into southern Europe. In contrast to *Ae. albopictus*, which has demonstrated superiority in competing for food resources in larval habitats in the US, especially in artificial container habitats, *Ae. japonicus* exhibits higher overwintering survival and earlier hatching. This biological advantage allows the larvae of *Ae. japonicus* to exploit habitat resources ahead of *Ae. albopictus* [27,28]. Although competition with the larvae of other *Aedes* species may influence the lifespan of adult *Ae. japonicus*, the utilization of diverse aquatic habitats mitigates the impact of such competition on the species’ success in new environments [29]. There are also suggestions that this species is outcompeting *Aedes atropalpus* (Coquillett, 1902) in certain US areas due to shorter larval development periods [30]. 

## 4. *Aedes* spp. Borne Diseases

### 4.1. Aedes japonicus as a Transmitter of Arboviruses and a Pest

In controlled laboratory environments, *Aedes japonicus* has exhibited competence as a vector for a diverse array of arboviruses, including chikungunya virus (CHIKV) [20], Cache Valley virus (CVV) [31], dengue virus (DENV) [20], eastern equine encephalitis virus (EEEV) [32], Japanese encephalitis virus (JEV) [33,34], La Crosse virus (LACV) [35], Rift Valley fever virus (RVFV) [36], St. Louis encephalitis virus (SLEV) [37], West Nile [15,38,39,40,41], and Zika virus (ZIKV) [31,42]. Although this broad spectrum of vector competence underscores the potential public health significance of *Ae. japonicus* in areas where it is present, it should be noted that individual studies on the vector competence of *Ae. japonicus* have been conducted under various experimental conditions, which could have influenced the results obtained. This is important, as there is no consensus for performing vector competence experiments. The presence of *Ae. japonicus* in North America and Europe has raised concerns, as wild-caught specimens have tested positive for the Cache Valley [43], La Crosse [44,45,46], and West Nile [47] viruses. This observation has led to the consideration of *Aedes japonicus* as a potential bridge vector, contributing to the transmission of these viruses to humans [13,48]. However, it is crucial to emphasize that additional field-collected evidence is imperative to delineate the specific role played by *Ae. japonicus* in these transmission cycles [49]. Table 1 summarizes bibliographic data regarding the competence of *Aedes japonicus* as a host (viruses detected in field-collected *Ae. japonicus* specimens) or laboratory vector for viruses.

The comparison presented in Table 1 indicates that, in Europe, there are local outbreaks of four diseases caused by viruses transmitted by *Ae. japonicus*, that is chikungunya, dengue, West Nile fever, and Zika. Local outbreaks of these diseases [52], in most cases, do not coincide with the current distribution range of *Ae. japonicus* in Europe [16], which may suggest that other invasive mosquito species like *Ae. albopictus* and *Ae. koreicus* (Edwards, 1917) or widely distributed indigenous European species, such as those from the *Culex pipiens* (Linnaeus, 1758) group, serve as vectors [52]. An exception is West Nile fever, where numerous local European outbreaks [53] overlap with the current distribution range of *Ae. japonicus*. However, even in this case, it has been demonstrated that the West Nile virus can be transmitted by native European species. In this context, *Culex torrentium* (Martini, 1925) should be considered one of the most important vector species from the *Culex pipiens* group, with very high transmission rates [54].

Notwithstanding its potential role in arbovirus transmission, *Aedes japonicus* has also been recognized as a mosquito pest in regions where it has established populations. Despite not being notably aggressive, its inclination to feed on human blood and its reproductive success in human-inhabited areas contribute to its potential as a nuisance species [14]. Understanding the ecological and epidemiological implications of *Ae. japonicus* in diverse settings is crucial for devising effective strategies to mitigate its impact on public health. 

### 4.2. Clinical Implications of Ae. japonicus-Transmitted Viruses

All the arboviruses listed in the previous section are RNA viruses. Table 2 outlines the main symptoms in humans caused by them [50,51]. A common characteristic for the majority is a low percentage of symptomatic patients among the infected, with particular concern regarding acute cases. Some of the mentioned diseases are rare or endemic in humans (Cache Valley virus disease, eastern equine encephalitis, and La Crosse encephalitis), while others, such as chikungunya, dengue, West Nile fever, and Zika, represent significant and growing burdens regarding febrile illnesses worldwide. For these reasons, the following sections will discuss the four latter diseases, as well as the current status of vaccinations against them.

*Ae. japonicus*-transmitted arboviruses pose a significant and escalating febrile illness burden worldwide. While severe clinical manifestations of dengue fever are less common, they can lead to hemorrhage, shock, and fatalities in some cases [55]. CHIKV infections are characterized by fever and severe arthralgias, with increasing evidence of fatal complications [56]. ZIKV attained the designation of a Public Health Emergency of International Concern in 2016 due to associations with congenital and neurological complications [57]. Co-infections are prevalent, and differential diagnosis is challenging in the absence of molecular diagnostics (e.g., PCR) because of similar clinical presentations [58,59,60,61,62,63]. No specific therapeutic treatment is available for these arboviruses, and vector control remains the primary public health intervention to prevent and respond to epidemics. While a DENV vaccine is available in some countries, it is recommended only for seropositive individuals older than nine years [64]. 

Temperature is a crucial factor in arbovirus transmission, exerting nonlinear effects on mosquito physiology and impacting the rates of development and mortality in *Aedes* spp. mosquitoes [65,66,67]. Additionally, temperature regulates viral incubation in the mosquito vector, with optimal warm temperatures reducing the extrinsic incubation period [63,64]. Understanding the effects of rainfall on arbovirus transmission is intricate and contingent on the local social–ecological context. The *Ae. aegypti* mosquito favors breeding and egg-laying in water-containing containers, often found in and around homes where female mosquitoes feed on people. Increased rainfall can lead to higher vector populations due to an abundance of rain-filled containers around dwellings [66]. Conversely, drought conditions and water scarcity can also boost vector populations if individuals begin storing water in containers around the home [67]. Additionally, temperature regulates viral incubation in the mosquito vector, with optimal warm temperatures reducing the extrinsic incubation period [63,64]. The influence of rainfall on arbovirus transmission is complex and contingent on the local social–ecological context. Numerous investigations have explored the relationships between temperature, *Aedes* spp. populations, and arbovirus transmission. Mechanistic models based on mosquito thermal biology suggest that DENV transmission occurs optimally at 28.1 °C in *Ae. aegypti* and 26.4 °C in *Ae. albopictus* [68]. Other studies have assessed the suitability of habitats for *Aedes* spp. mosquito expansion and the potential of regions to support arbovirus transmission under current climate conditions and predicted climate change scenarios [69,70,71]. For example, a study in Ecuador projected the expansion of *Ae. aegypti* into higher elevations of the Andean foothills, estimated to affect 4215 km^2^ of new territory and 12,000 people under the most extreme climate change scenario by 2050 [72]. Such elevational range expansions are anticipated throughout the tropical *Aedes* spp., increasing the population at risk of arboviral infections. While the global burden of arboviral diseases is expected to worsen, some models suggest that the transmission of arboviruses may decrease in tropical regions due to temperatures becoming too warm to sustain mosquito populations [68,69]. Additionally, local mosquito populations may evolve to be better adapted to changing climate conditions. 

#### 4.2.1. Expansion of Dengue 

Dengue virus (DENV) resurfaced in the Americas during the 1980–1990 decade, with simultaneous circulation of all four serotypes belonging to the family Flaviviridae and the genus *Flavivirus* (DENVs 1–4), precipitating significant urban epidemics throughout the region [73]. Over the past two decades, dengue has extended its geographic range beyond subtropical and tropical areas, manifesting local transmission and outbreaks in diverse regions of the United States, southern Europe, Uruguay, Argentina, and Australia [74,75,76,77,78,79,80]. Outbreaks have been observed in Hawaii, southern Florida, and along the Mexico border in the United States [74,75,78,81]. In Europe (2012–2013), a notable outbreak occurred in Portugal (Madeira). It is believed that DENV originated in Venezuela [77,82]. Local transmission was also documented for the first time (2010) in southern Croatia and France [76,79], and in the following years, local outbreaks of dengue were also reported in Spain and Italy [50,52,79]. In Italy, in 2023, 82 autochthonous cases were reported, which is a record value for Europe [79]. In South America, autochthonous dengue transmission was documented in subtropical regions of Argentina in 1997 and Uruguay in 2016, marking the first instances since *Ae. aegypti* was thought to have been eradicated in the 1960s [80,83]. Nowadays, *Aedes* spp. populations are widespread throughout Europe, temperate South America, and the United States. It could be an escalating threat due to heightened global travel and a climate conducive to transmission. In endemic areas, dengue outbreaks are increasing in frequency and severity. In 2019, tropical and subtropical regions experienced a significant surge in dengue transmission, with the Americas witnessing a resurgence of DENV. The Pan American Health Organization (PAHO) reported 3,139,335 cases of dengue fever in 2019 alone, marking the highest number ever reported in a single year in the Americas [84]. Brazil alone accounted for over two million cases, with the highest incidence rates observed in Belize, Nicaragua, and Honduras [84]. 

#### 4.2.2. Chikungunya and Zika: A Growing Threat

In 2013, chikungunya (CHIKV) emerged in the Caribbean and rapidly spread throughout Central and South America [85]. Both *Ae. aegypti* and *Ae. albopictus* were implicated in the transmission of CHIKV [86]. The expansion of both vectors due to meteorological extremes likely played a role in the rapid spread of chikungunya, with numerous outbreaks following extreme rainfall events [87,88]. The year 2014 marked the first in a sequence of years listed as the warmest on record [89]. A substantial outbreak of chikungunya was reported in Italy, with over 300 cases, and autochthonous transmission was also reported in France [90,91]. In the Americas, over 1.1 million cases were reported in 2014 [92]. Starting in 2015, Zika (ZIKV) emerged and spread intensely throughout the Americas. Between 2015 and 2016, laboratory-confirmed and suspected cases were reported from 87 countries and territories worldwide, with over 750,000 cases reported from the Americas alone [92]. Autochthonous ZIKV transmission has been reported in southern Florida, France, and Argentina. 

#### 4.2.3. West Nile Fever Expansion 

West Nile virus (WNV) was first described in 1937 in the West Nile district of Uganda [93]. It has been shown that WNV circulates in natural cycles between mosquitoes and birds, while humans and other mammals, such as horses, are incidental hosts. Although the majority of infections occur without symptoms, approximately 20% of those infected develop fever, and acute consequences such as encephalitis or meningitis, occurring in 1 out of 150 individuals, can be life-threatening. Since its discovery, outbreaks of West Nile fever have been recorded in the Middle East, Africa, and Asia, and in the 21st century, increasingly in the US and Europe [94,95,96]. In 2023, EU/EEA countries reported 707 cases of WNV infection in humans, mainly in Italy, Greece, Romania, France, and Hungary, but cases were also detected in Spain, Germany, Croatia, and Cyprus [97]. Currently, the northern range of West Nile virus occurrence is Germany, where WNV has been detected since 2018 both in birds and equines, as well as in humans [98]. Its presence in other neighboring countries, e.g., the Czech Republic [98], leads to the assumption that it also occurs in Poland, although no local outbreaks of the disease have been detected here so far. Although West Nile fever outbreaks occur with unpredictable frequency and location, there has been a gradual expansion of the disease’s geographical range in Europe.

### 4.3. Integrated Strategies for Aedes spp.-Borne Viral Infections: Vaccination and Vector Control

Effective control of *Aedes* spp.-borne viral infections has been demonstrated through various strategies, including preventive measures like maintaining environmental cleanliness and proper drainage; minimizing contact with vectors; vaccinating susceptible individuals; and employing genetic control of mosquitoes, including paratransgenesis. These multifaceted approaches contribute to the reduction in disease burden associated with *Aedes* spp.-transmitted viruses [99]. Control strategies for mosquito-borne infections transmitted by *Aedes* spp. mosquitoes include reducing human susceptibility through vaccinations. Effective vaccines, such as those for yellow fever and Japanese encephalitis, exist, although the safety of the dengue vaccine remains controversial. Ongoing research also focuses on developing vaccines for other *Aedes* spp.-borne viruses, such as ZIKV and CHIKV [100]. Preventive measures for both humans and vectors aim to minimize contact between individuals and infected mosquitoes. These measures encompass the use of mosquito nets, repellents, and the promotion of a clean environment and proper drainage. The application of metofluthrin in homes has proven effective in reducing mosquito biting frequency and population density. Vector control strategies aim to decrease the abundance and transmission capacities of virus-carrying mosquitoes, but their implementation faces challenges [100]. These approaches, crucial for suppressing dengue transmission, often require substantial financial and labor investments. Some methods, such as the use of chemical larvicides, carry the risk of environmental contamination [100]. 

#### Vaccination Status

- Chikungunya vaccine

On 9 November 2023, the FDA approved the first chikungunya vaccine [101]. Ixchiq is administered as a solitary injection into the muscle, constituting a live, attenuated form of the chikungunya virus. The vaccine has the potential to induce symptoms in the recipient akin to those observed in individuals afflicted with chikungunya disease [101]. The safety profile of Ixchiq underwent assessment through two clinical studies conducted in North America, encompassing approximately 3500 participants aged 18 and above who received the vaccine. One of the studies incorporated about 1000 participants who were administered a placebo. Among the reported side effects in vaccine recipients, the most frequently encountered were headache, fatigue, muscle pain, joint pain, fever, nausea, and tenderness at the injection site. Furthermore, while not a common occurrence, severe chikungunya-like adverse reactions were documented in 1.6% of Ixchiq recipients, hindering daily activities and/or necessitating medical intervention. Two recipients with severe chikungunya-like adverse reactions required hospitalization. Additionally, some recipients experienced prolonged chikungunya-like adverse reactions persisting for at least 30 days. The prescribing information includes a cautionary warning regarding the potential for the vaccine to induce severe or prolonged chikungunya-like adverse reactions [101].

- Zika vaccine

Many types of vaccines against ZIKV are under development. Viral vector vaccines, particularly adenovirus (Ad) vectors such as Ad5-prM-E and Ad26.ZIKV.M-Env, have demonstrated strong and durable immune responses against Zika virus in preclinical models. Ad5-prM-E, in particular, has shown superior protection compared with Ad4-prM-E in a mouse model. Additionally, a chimpanzee adenoviral vector (ChAdOx1) and poxvirus-based vectors, like MVA-ZIKV, have shown promise in inducing immune responses and reducing viremia in animal models, supporting their potential as Zika vaccine candidates. Poxvirus-based vectors, exemplified by Modified Vaccinia Ankara (MVA) and Sementis Copenhagen Vector (SCV), offer attractive features for vaccine development, including a large payload capacity and the ability to induce both cellular and humoral immunity. Further studies and optimizations, such as dose adjustments, may enhance the efficacy of these candidates [102].

The DNA vaccine platform, employed for over twenty-five years, has successfully generated candidate vaccines against various pathogens, including West Nile virus, Ebola virus, and SARS-CoV-2. By cloning pathogen antigens into DNA plasmids, DNA vaccines offer a safe and immunogenic approach, inducing both cellular and humoral responses. Notably, a DNA vaccine encoding consensus ZIKV premembrane and envelope antigens demonstrated efficacy, providing complete protection against ZIKV in mice. The platform’s advantages include rapid design, low cost, stability, and safety for adults and fetuses. Additionally, the Vaccine Research Center developed the VRC5288 and VRC5283 DNA vaccine candidates, which completed clinical trials and have shown promise in non-human primates. The DNA vaccine GLS-5700, targeting a synthetic ZIKV prM-E antigen, provides protection to mice against ZIKV-induced morbidity, mortality, and testicular damage, highlighting the platform’s potential in addressing emerging infectious diseases, including Zika outbreaks [102].

mRNA vaccine technology, known for its simplicity, flexibility, and rapid production, has become a versatile platform for vaccine development. The main advantage lies in the swift modification of mRNA-based vaccines to eliminate undesired side effects or enhance immunogenicity, responding effectively to mutations and antigenic changes. A ZIKV vaccine platform utilizing lipid nanoparticles to encapsulate modified mRNA (mRNA-LNP) encoding ZIKV structural genes has demonstrated efficacy in producing virus-like particles and generating high levels of neutralizing antibodies, providing protection in both immunocompetent and immunocompromised mice. Another mRNA vaccine candidate, encoding ZIKV Brazil strain SPH2015 prM-E glycoproteins, induced protective antibody responses in mice, highlighting its potential as a promising vaccine candidate. The success of mRNA technology in addressing the COVID-19 pandemic has further prompted the development of mRNA vaccines against ZIKV and other infectious diseases as a promising alternative to traditional vaccine approaches. Moderna’s Zika vaccine candidate (mRNA-1893) has shown promising results in preclinical studies and is currently undergoing phase II clinical trials in collaboration with the Biomedical Advanced Research and Development Authority (BARDA) [102].

- Dengue vaccine

The dengue vaccine is designed to prevent dengue fever in humans. Efforts to develop such vaccines began in the 1920s, but progress was hindered by the challenge of creating immunity against all four dengue serotypes [103]. Currently, as of 2023, two commercially available vaccines are Dengvaxia and Qdenga [104]. Dengvaxia is recommended for individuals who have previously experienced dengue fever or populations with widespread prior infections. However, its value is limited because it may increase the risk of severe dengue in those who have not been previously infected [104,105]. Controversy arose in 2017 when over 733,000 children and 50,000 adults were vaccinated with Dengvaxia, regardless of their serostatus [106]. On the other hand, Qdenga is designated for individuals who have not been previously infected [107]. Furthermore, there are ongoing efforts to develop other vaccine candidates, which include live attenuated, inactivated, DNA, and subunit vaccines.

- West Nile virus vaccine

Since the late 1990s, extensive research has focused on creating vaccines for West Nile virus in both human and veterinary contexts [108]. While equine vaccines have achieved licensure success, human immunization remains elusive. The quest for an effective WNV vaccine has embraced a multifaceted approach, employing diverse technological platforms such as recombinant proteins, virus-like particles, RNA-replicons, chimeric flaviviruses, and genetic vaccines (viral vectors expressing WNV genes; DNA- and RNA vaccines); however, it has not brought satisfactory results [108]. 

Some of the vaccine candidates that have proven to be protective in animal models have been transferred to clinical testing in humans. West Nile fever is most severe in the elderly; hence, the major target population for this vaccine has an aged immune system. Vaccine candidates tested so far have demonstrated high immunogenicity in individuals >50 years of age. The effectiveness of existing vaccine candidates is also controversial, and usually, three doses of vaccine are required to achieve a relatively high level of immunization [107].

- Challenging vaccines for DENV, ZIKV, CHIKV, and WNV

Arboviruses, including DENV, ZIKV, CHIKV, and WNV, are primarily transmitted by arthropods. A novel strategy to prevent arboviral diseases involves recognizing the role of arthropod saliva in facilitating pathogen transmission. Arthropod-borne viruses share mosquitoes and ticks as common vectors. Arthropod saliva components aid viral pathogenesis by exploiting the host’s immune responses, making vaccines targeting mosquito salivary proteins a promising approach. Salivary proteins from key vector mosquitoes, such as *Ae. aegypti*, could contribute to protection against multiple mosquito-borne viral infections. A specific salivary gland protein (NeSt1) stimulates neutrophils, altering the immune microenvironment and enhancing early ZIKV replication. Vaccination experiments in mice suggest a NeSt1-based vaccine could protect against severe Zika virus infection. Another salivary protein, AgBR1, induces inflammatory responses at the bite site. Passive and active immunization with AgBR1 has partially protected mice from mosquito-borne ZIKV infection, making AgBR1 a potential vaccine target. Combining AgBR1- and NeSt1-sera in passive immunization has enhanced survival and reduced viral burden, protecting against mosquito-borne ZIKV infection. Targeting a combination of mosquito saliva proteins emerges as a promising approach for vaccine development against mosquito-borne ZIKV infection [102]. 

## 5. Conclusions

The identification of *Aedes japonicus* in Poland marks a significant expansion of Europe’s invasive mosquito species, raising concerns about potential disease transmission given its vector competence for the dengue, chikungunya, Zika, and West Nile viruses. This discovery highlights the urgent need for proactive measures and international collaboration to mitigate the impact on public health. In conclusion, the initial identification of *Aedes japonicus* in Poland serves as a catalyst for further studies and emphasizes the importance of monitoring and controlling invasive mosquito species. The global implications of these findings underscore the need for continued research and collaborative efforts to safeguard public health against emerging vector-borne diseases. This research emphasizes the importance of systematically studying mosquitoes in their natural habitats to comprehend the intricate relationship between vector populations and arbovirus transmission. The knowledge gained from this investigation will aid in the development of evidence-based strategies to mitigate the public health risks associated with mosquito-borne diseases in Europe.

It seems that, at the moment, considerations regarding the role of *Ae. japonicus* as a vector of CHIKV, DENV, WNV, and ZIKV in Europe are speculative. It should be noted, however, that both the level of knowledge of local disease outbreaks caused by these arboviruses in Europe and the specificity of field faunistic data regarding the actual distribution range of *Ae. japonicus* are undoubtedly incomplete. Confounding factors in this respect may include, on the one hand, the characteristic asymptomatic nature prevalent in most infected individuals or the improper diagnosis of the disease due to nonspecific symptoms in severe cases, as well as insufficient funding for basic scientific research in the field of faunistic studies. Regardless of the above questions, although available data indicate a territorial mismatch between disease outbreaks and the current distribution range of *Ae. japonicus* in Europe (the exception being the range of West Nile virus, which overlaps with the range of *Ae. japonicus*), it is not difficult to imagine a scenario in which these ranges overlap, especially in the context of the rapidly increasing range of occurrence of *Ae. japonicus*. 

## Figures and Tables

**Table 1:** Host and vector competence of *Aedes japonicus*.

Virus		Outbreaks of Disease [50,51]	*Ae. japonicus* as a	
Full Name	Abbreviation		Host *	Vector **
Chikungunya virus	CHIKV	Africa (Central and South Africa countries; Madagascar), Asia (the Arabian Peninsula, South Asia, China, and the Malay Archipelago), Central and South America, the US, and some European countries (Italy; France)		[20]
Cache Valley virus	CVV	US (North Carolina, Missouri, Wisconsin, and New York)	[43]	[31]
Dengue virus	DENV	Africa (Central and South Africa countries, Northeast Africa, and Madagascar), Asia (the Arabian Peninsula, South Asia, and the Malay Archipelago), Central and South America, the US (Virgin Islands), European countries (Croatia, Italy, France, Spain, and Madeira), and Northeast Australia		[20]
Eastern equine encephalitis virus	EEEV	North America (Canada, USA) and South America (northern and central parts of North America)		[32]
Japanese encephalitis virus	JEV	Asia (South and Eastern Asia, the Japanese archipelago, China, Russia, and the Malay Archipelago), Australia, and Oceania		[33,34]
La Crosse virus	LACV	US (Midwestern, Southwestern, and Southeastern United States)	[44,45]	[35]
Rift Valley fever virus	RVFV	Africa (Central and South Africa countries, Northeast Africa, and Madagascar), Asia (the Arabian Peninsula)		[36]
St. Louis encephalitis virus	SLEV	USA (Michigan, California, Arizona, and the Southeastern United States)		[37]
West Nile virus	WNV	Almost all of Africa, with Madagascar; Europe (Croatia, Cyprus, France, Germany, Greece, Hungary, Italy, North Macedonia, Romania, Serbia, and Spain); Asia (the Arabian Peninsula, Southwest Asia, China, and Russia); North America (USA; Canada); Central and South America; Australia; and Oceania	[47]	[15,38,39,40,41]
Zika virus	ZIKV	Africa (Central and South Africa countries, Northeast Africa, and Madagascar), Asia (the Arabian Peninsula, South Asia, China, Russia, and the Malay Archipelago), North America (US), Central and South America, Europe (France), Australia, and Oceania		[31,42]

* Virus detected in field-collected *Ae. japonicus* specimens; ** *Ae. japonicus* as a competent laboratory vector.

**Table 2:** Clinical signs and symptoms of infection with pathogens transmitted by *Ae. japonicus* [50,51].

Viral Pathogen	Clinical Signs and Symptoms in Humans
Chikungunya	Most subjects infected with CHIKV will develop some symptoms. The most common symptoms are fever and joint pain. Other symptoms may include muscle pain, headache, nausea, fatigue, and rash. Serious complications are rare (eye, nervous system, heart, and gastrointestinal complaints). The risk of severe disease is increased in newborns; adults over 65 years; and people with high blood pressure, diabetes, or heart disease. May be a cause of death in older people.
Cache Valley virus	Initial symptoms are fever, headache, nausea, vomiting, fatigue, and rash. Severe CVV infection may lead to encephalitis and/or meningitis, with symptoms like stiff neck, confusion, loss of coordination, difficulty speaking, and seizures. A severe form may lead to death. Relatively few cases of the disease have been identified to date.
Dengue	One in four infected people show symptoms. Among the sick, one in twenty people show severe symptoms. Rarely, severe dengue can lead to death. Mild symptoms may include high fever, severe headache, pain behind the eyes, muscle and joint pains, nausea, vomiting, swollen glands, and rash. Severe dengue symptoms are severe abdominal pain, persistent vomiting, rapid breathing, bleeding gums or nose, fatigue, restlessness, blood in vomit or stool, strong thirst, pale and cold skin, and general weakness.
Eastern equine encephalitis	Most infected subjects do not develop symptoms. EEE can result in febrile illness or neurologic disease. Febrile illness is characterized by fever, chills, body aches, and joint pain. Symptoms of neurologic disease are headache, vomiting, diarrhea, seizures, behavioral changes, drowsiness, coma, and long-term physical or mental impairments (from mild brain dysfunction to severe intellectual impairment, personality disorders, seizures, paralysis, and cranial nerve dysfunction). One-third of cases with encephalitis due to EEE die. Very rare in humans.
Japanese encephalitis	Mild or no symptoms in most cases. In some cases, they are headache, fever, disorientation, seizures, weakness, coma, and encephalitis (1/4 cases with encephalitis due to JEV are fatal).
La Crosse encephalitis	Most infected subjects do not have symptoms. Initial symptoms are fever, headache, nausea, vomiting, tiredness, and lethargy (reduced activity or alertness). Severe disease (most frequent in children aged <16 years) may include encephalitis with symptoms like high fever, headache, neck stiffness, stupor, disorientation, coma, tremors, seizures, muscle weakness, vision loss, numbness, and paralysis. Death does occur rarely (<1%).
Rift Valley fever	Usually, no symptoms or a mild illness (fever, weakness, back pain, and dizziness at the onset of illness). About 8–10% of patients develop much more severe symptoms, including ocular disease (from mild to vision loss); encephalitis (including headaches, coma, or seizures); and hemorrhagic fever, which occurs in less than 1% of all RVF patients (vomiting blood, bloody stool, or bleeding with fatality of around 50%).
St. Louis encephalitis	A lack of symptoms in most people. In some cases, symptoms include fever, headache, dizziness, nausea, and generalized weakness. In severe cases, encephalitis or meningitis, stiff neck, confusion, disorientation, dizziness, tremors, unsteadiness, and coma occur. The risk of death is approximately 5–20% and increases with age.
West Nile fever	Infection with WNV is asymptomatic in around 80% of infected subjects. About 20% will develop West Nile fever (fever, headache, tiredness, body aches, nausea, and vomiting, occasionally with a skin rash on the trunk and swollen lymph glands). Severe consequences (1 in 150 infected subjects) are encephalitis, meningitis, or poliomyelitis (with symptoms such as headache, high fever, neck stiffness, stupor, disorientation, coma, tremors, convulsions, muscle weakness, and paralysis). There is a higher risk of severe symptoms in people over 60 years of age, with cancer, diabetes, hypertension, and kidney disease, and in people who have received organ transplants. One in ten people with severe symptoms die.
Zika	Mild or no symptoms in most cases. Mild symptoms are fever, rash, conjunctivitis, muscle and joint pain, malaise, and headache. There are consequences of infection during pregnancy: microcephaly and other congenital abnormalities, fetal loss, stillbirth, and preterm birth. In adults and older children, Guillain–Barré syndrome, neuropathy, and myelitis.

## Data Availability

Not applicable.
